# Astragaloside IV Attenuates Podocyte Apoptosis Mediated by Endoplasmic Reticulum Stress through Upregulating Sarco/Endoplasmic Reticulum Ca^2+^-ATPase 2 Expression in Diabetic Nephropathy

**DOI:** 10.3389/fphar.2016.00500

**Published:** 2016-12-21

**Authors:** Hengjiang Guo, Aili Cao, Shuang Chu, Yi Wang, Yingjun Zang, Xiaodong Mao, Hao Wang, Yunman Wang, Cheng Liu, Xuemei Zhang, Wen Peng

**Affiliations:** ^1^Laboratory of Renal Disease, Putuo Hospital, Shanghai University of Traditional Chinese MedicineShanghai, China; ^2^Department of Nephrology, Putuo Hospital, Shanghai University of Traditional Chinese MedicineShanghai, China; ^3^Experimental Research Center, Putuo Hospital, Shanghai University of Traditional Chinese MedicineShanghai, China; ^4^Department of Pharmacology, School of Pharmacy, Fudan UniversityShanghai, China

**Keywords:** astragaloside IV, diabetic nephropathy, sarco/endoplasmic reticulum Ca^2+^-ATPase 2, endoplasmic reticulum stress, podocyte apoptosis

## Abstract

Sarco/endoplasmic reticulum Ca^2+^-ATPase (SERCA) plays a central role in the pathogenesis of diabetes. This protein has been recognized as a potential target for diabetic therapy. In this study, we identified astragaloside IV (AS-IV) as a potent modulator of SERCA inhibiting renal injury in diabetic status. Increasing doses of AS-IV (2, 6, and 18 mg kg^-1^ day^-1^) were administered intragastrically to *db/db* mice for 8 weeks. Biochemical and histopathological approaches were conducted to evaluate the therapeutic effects of AS-IV. Cultured mouse podocytes were used to further explore the underlying mechanism *in vitro*. AS-IV dose-dependently increased SERCA activity and SERCA2 expression, and suppressed ER stress-mediated and mitochondria-mediated apoptosis in *db/db* mouse kidney. AS-IV also normalized glucose tolerance and insulin sensitivity, improved renal function, and ameliorated glomerulosclerosis and renal inflammation in *db/db* mice. In palmitate stimulated podocytes, AS-IV markedly improved inhibitions of SERCA activity and SERCA2 expression, restored intracellular Ca^2+^ homeostasis, and attenuated podocyte apoptosis in a dose-dependent manner with a concomitant abrogation of ER stress as evidenced by the downregulation of GRP78, cleaved ATF6, phospho-IRE1α and phospho-PERK, and the inactivation of both ER stress-mediated and mitochondria-mediated apoptotic pathways. Furthermore, SERCA2b knockdown eliminated the effect of AS-IV on ER stress and ER stress-mediated apoptotic pathway, whereas its overexpression exhibited an anti-apoptotic effect. Our data obtained from *in vivo* and *in vitro* studies demonstrate that AS-IV attenuates renal injury in diabetes subsequent to inhibiting ER stress-induced podocyte apoptosis through restoring SERCA activity and SERCA2 expression.

## Introduction

Diabetic nephropathy remains the most common microvascular complication of diabetes and the leading cause of end-stage-renal disease (ESRD) worldwide. Recently, accumulating evidence have indicated that podocyte apoptosis plays an important role in the pathogenesis of DN (Reidy et al., 2014). However, the underlying mechanism is not completely understood.

Endoplasmic reticulum is a cellular organelle that is responsible for protein processing. Some pathophysiological stress leads to the accumulation of aberrant unfolded proteins in the ER lumen, which in turn initiates a well-conserved signaling cascade called the UPR to mitigate ER stress through the mediation of three ER-resident transducers: activating transcription factor 6 (ATF6), PERK, and IRE1 (Inagi, 2010; Inagi et al., 2014). However, under prolonged or excessive ER stress, the apoptotic signaling will be induced, leading to cell injury and death through the mediation of downstream molecules, such as CHOP, c-Jun N-terminal kinases (JNK), and caspase 12 (Inagi, 2010; Inagi et al., 2014). Recently, ER stress has emerged as one of the central mechanisms that lead to diabetic complications and inhibition of ER stress improves diabetic symptoms (Ozcan et al., 2006; Qi et al., 2011). Excessive ER stress results in podocyte apoptosis while suppression of ER stress attenuates podocyte apoptosis *in vivo* and *in vitro* (Chen et al., 2008; Cao et al., 2016).

In addition to being a major intracellular storage site for calcium (Ca^2+^), ER maintains its normal function depending heavily on intraluminal calcium concentrations (Ashby and Tepikin, 2001; Vangheluwe et al., 2005). Perturbation of ER Ca^2+^ homeostasis leads to ER stress and the activation of UPR (Ron and Walter, 2007; Ozcan and Tabas, 2012). The SERCA, which pumps cytosolic Ca^2+^ into the ER, is an imperative maintainer of ER Ca^2+^ homeostasis (Ashby and Tepikin, 2001; Vangheluwe et al., 2005). The mammalian SERCA family is comprised of three tissue-specific members, SERCA1-3, with SERCA2 being the most widespread isoform (Ashby and Tepikin, 2001; Vangheluwe et al., 2005). Several investigations have revealed a loss of SERCA2 isoform b (SERCA2b) expression and activity in islets (Cardozo et al., 2005; Kono et al., 2012), heart (Wold et al., 2005; Takada et al., 2012), and liver (Park et al., 2010; Fu et al., 2011) in selected models of diabetes, suggesting that SERCA2 dysfunction is a potential pathology for development of diabetic complications. Disruption of ER Ca^2+^ homeostasis caused by impaired activity or expression of SERCA2 triggers ER stress (Cardozo et al., 2005; Fu et al., 2011), while increasing SERCA2 function by SERCA2 overexpression or SERCA2 activators alleviates ER stress and improves diabetic conditions (Park et al., 2010; Kang et al., 2015).

Saponin AS-IV is one of the active components of Astragalus membranaceus (Fisch) Bge, which has been shown to possess comprehensive pharmacological activities in treating renal diseases (Rios and Waterman, 1997; Peng et al., 2008). A body of studies have addressed the renoprotective role of AS-IV, including suppressing renal inflammation (Gui et al., 2013), inhibiting renal tubulointerstitial fibrosis (Wang et al., 2014a,b), and protecting podocytes (Gui et al., 2012; Chen et al., 2014a). Recent investigations show that AS-IV attenuates proteinuria and podocyte apoptosis in streptozotocin-induced DN via the inhibition of ER stress (Chen Y. et al., 2014; Wang et al., 2015), but the underlying mechanism needs to be further elucidated. The reports that SERCA2b is a major regulator of ER stress (Park et al., 2010) and AS-IV can modulate SERCA2a expression in myocardial injury (Xu et al., 2007, 2008) prompt us to test whether AS-IV alleviates ER stress through regulating SERCA. Therefore, the current study is undertaken to define whether SERCA2 is implicated in the renoprotective effect of AS-IV in *db/db* mice, a mouse model of type 2 diabetes, and palmitate-stimulated mouse podocyte cell line.

## Materials and Methods

### Drugs

Astragaloside IV was purchased from Shanghai Bogoo Biotechnology Company, Limited (purity at 98%, Shanghai, China). RGZ was purchased from Sigma-Aldrich (St. Louis, MO, USA).

### Animals and Drug Administration

*db/db* mouse exhibits clinical and histological features of DN resembling those found in human DN, such as hyperglycemia, hyperinsulinemia, hyperlipidemia, obesity, albuminuria, glomerular enlargement, and mesangial matrix expansion (Sharma et al., 2003; Tesch and Lim, 2011). Six-week-old male diabetic *db/db* (BKS.cg-m +/+ Leprdb/J) and age-matched non-diabetic *db/m* littermates were purchased from Model Animal Research Center of Nanjing University (Nanjing, China). All the work was carried out in accordance with the approved guidelines for the use of experimental animals in Putuo Hospital, Shanghai University of Traditional Chinese Medicine. The BKS.cg-m +/+ Leprdb/J mouse becomes obese and diabetic by 8 weeks of age. They were housed in Experimental Animal Facilities at Shanghai Putuo District Central Hospital under specific-pathogen-free (SPF) conditions. Animals were fed with standard diet and free to water. At 8 weeks of age, *db/m* and *db/db* mice were randomly assigned to seven groups (*n* = 10/each group): (1) normal *db/m* mice receiving vehicle (*db/m*-vehicle), (2) *db/m* mice receiving AS-IV at 18 mg kg^-1^ day^-1^ (*db/m*-18 mg kg^-1^ day^-1^ AS-IV), (3) diabetic control *db/db* mice receiving vehicle (*db/db*-vehicle), (4) *db/db* mice receiving AS-IV at 2 mg kg^-1^ day^-1^ (*db/db*-2 mg kg^-1^ day^-1^ AS-IV), (5) *db/db* mice receiving AS-IV at 6 mg kg^-1^ day^-1^ (*db/db*-6 mg kg^-1^ day^-1^ AS-IV), (6) *db/db* mice receiving AS-IV at 18 mg kg^-1^ day^-1^ (*db/db*-18 mg kg^-1^ day^-1^ AS-IV), and (7) *db/db* mice receiving RGZ as the positive control at 2 mg kg^-1^ day^-1^ (*db/m*-2 mg kg^-1^ day^-1^ RGZ). AS-IV and RGZ were suspended in 0.5% carboxymethyl cellulose as a vehicle for their administrations and were given to mice by oral gavage once daily for 8 weeks. Dose conversion from human to animal was using the body surface area normalization method (Reagan-Shaw et al., 2008).

### Measurement of Metabolic and Physiological Parameters

Body weight, food and water intake, urine volume, fasting blood glucose, and systolic blood pressure were measured at 4-week intervals. Mice were housed in individual metabolic cages for 24 h urine collection. Fasting blood glucose levels were monitored with the Omron HEA-230 Glucometer using one drop of tail blood. Systolic blood pressure was obtained by tail-cuff plethysmography using the ALC-NIBP blood pressure measuring system (Shanghai Alcott Biotech Co., Ltd., Shanghai, China). At the end of the study, blood samples were drawn from orbit and centrifuged for serum collection. Then the animals were killed and both kidneys were removed immediately. A portion of the renal cortex was fixed in 4% paraformaldehyde for histological examination. The rest of the renal cortex was snap-frozen in liquid nitrogen and then stored at -80°C for protein and total RNA extraction, and for ER isolation. Urinary albumin (albuminuria) was measured using an ELISA Kit (Biovision, Milpitas, CA, USA) and urinary albumin excretion was expressed as urinary albumin to creatinine ratio (ACR). BUN was measured using Urea Nitrogen Colorimetric Detection Kit (Arbor Assays, Ann Arbor, MI, USA). Urinary and serum creatinine levels were determined by Creatinine Colorimetric/Fluorometric Assay Kit (Biovision, Milpitas, CA, USA). Serum HbA1c was measured using Mouse Glycated Hemoglobin (HbA1C) ELISA Kit (Wuxi Donglin Sci &Tech Development Co., Ltd., Wuxi, China). Serum insulin was measured using Rat/Mouse Insulin 96 Well Plate Assay Kit (Millipore, Billerica, MA, USA). All the biochemical parameters were determined according to the manufacturer’s instructions. Insulin resistance was determined by calculating the HOMA-IR. HOMA-IR index was calculated according to the formula: HOMA-IR (mmol/L × μU/mL) = [fasting glucose (mmol/l)] × [fasting insulin (μU/ml)]/22.5 (He et al., 2014).

### Oral Glucose Tolerance Test (OGTT) and Intraperitoneal Insulin Tolerance Test (IPITT)

After 8 weeks of treatment, mice were fasted for 6 h (8:00 AM to 2:00 PM), and the oral glucose tolerance test (OGTT) and intraperitoneal insulin tolerance test (IPITT) experiments were performed as previously described (Chen et al., 2014b; Wang C. et al., 2014). Briefly, the animals were orally administered with glucose (2 g kg^-1^ body weight) or intraperitoneally administrated with insulin (0.75 U kg^-1^ body weight), and blood glucose levels were monitored over a 2 h period using Omron Glucometer. Quantification of AUC was achieved using Graphpad Prism software 5 software (GraphPad Software Inc., San Diego, CA, USA).

### Renal Histology and Immunohistochemistry

Renal cortex was processed, embedded in paraffin and cut into 5 μm-thick sections. Renal sections were stained with PAS. Semiquantitative scoring of glomerular sclerosis was performed in a blinded manner using a five-grade method described previously (Taneda et al., 2003). Twenty to thirty glomeruli randomly selected from per mouse were scored from six mice in each group. For immunohistochemistry, the sections were incubated with primary antibody against Wilms’ tumor-1 (WT-1), Podocin, monocyte chemotactic protein-1 (MCP-1), GRP78 (Abcam, Cambridge, MA, USA), TNF-α (Santa Cruz Biotechnology, Santa Cruz, CA, USA) overnight at 4°C, and then incubated with Biotinylated secondary antibody (Vector Laboratories, Burlingame, CA, USA) followed by VECTASTAIN ABC Reagent (Vector Laboratories, Burlingame, CA, USA) incubation for signal amplification. Color development was achieved using 3,3′-diaminobenzidine (DAB) (Vector Laboratories, Burlingame, CA, USA). The IOD was assessed by computer analysis with Image-Pro plus 6.0 (Media Cybernetics, MD, USA).

### TUNEL Staining

TUNEL staining was performed with the ApopTag Plus Peroxidase *In Situ* Apoptosis Detection Kit (Millipore, Billerica, MA, USA) according to the manufacturer’s instructions. TUNEL-positive cells were semiquantified by randomly counting at least 30 glomeruli in each mouse.

### Western Blotting

Renal cortex or cultured podocytes were lysed with the buffer containing 50 mM Tris (pH7.5), 150 mM NaCl, 1% Triton X-100, 0.5% sodium deoxycholate, 2 mM EDTA, 0.5 mM dithiothreitol, 1 mM PMSF, protease inhibitor cocktail and phosphatase inhibitor (both from Sigma-Aldrich, St. Louis, MO, USA). Proteins were separated by SDS-PAGE and western blotting was carried according to the standard procedures. The following primary antibodies were used: eIF2α, JNK, phospho-JNK^Thr183/Tyr185^, AIF, cyclophilin D, and ANT from Santa Cruz Biotechnology (Santa Cruz, CA, USA), GAPDH, SERCA1, inositol-requiring enzyme 1α (IRE1α), TNF receptor associated factor 2 (TRAF2), PERK, phospho-PERK^Thr980^, phospho-eIF2α^Ser51^, activating transcription factor 4 (ATF4), caspase 12, caspase 9, caspase 3, CHOP, and cytochrome *c* from Cell Signaling Technology (Danvers, MA, USA), SERCA2, Podocin, Nephrin, GRP78, ATF6, XBP1, voltage dependent anion channel 1 (VDAC1), APAF1, and PPARγ from Abcam company (Cambridge, MA, USA), phospho-IRE1α^Ser724^ from Novus Biologicals, SERCA3 from Proteintech Group (Chicago, IL, USA), ASK1 from ProSci Incorporated (San Diego, CA, USA). Secondary antibodies were horseradish peroxidase-conjugated goat anti-rabbit IgG (BOSTER, Wuhan, China) and goat anti-mouse IgG (BOSTER, Wuhan, China), and signals were detected using Immobilon Western Chemiluminescent HRP Substrate (Millipore, Billerica, MA, USA). Densitometric quantitation was performed using Image J 1.37 software (NIH, Bethesda, MD, USA). Protein expression was quantified as the ratio of specific band to GAPDH.

### Quantitative RT-PCR (qRT-PCR)

Total RNAs were isolated using Trizol reagents (Invitrogen, Carlsbad, CA, USA). First-strand cDNA was synthesized from 1 μg total RNA in a 20 μl reaction volume using a random primer (Takara, Dalian, China) and Moloney murine leukemia virus reverse transcriptase (New England Biolabs, Ipswich, MA, USA). SYBR green based qRT-PCR was performed in an Applied Biosystems ViiA 7^TM^ real time PCR system using the following thermal cycle reaction protocol: 50°C for 2 min, 95°C for 10 min, then 40 cycles at 95°C for 15 s, and at 60°C for 1 min. Primers used to amplify SERCA genes were used as previously described (Kono et al., 2012) and synthesized by Sangon Biotech (Shanghai) Co., Ltd. (Shanghai, China). The relative mRNA amount was normalized to the invariant β-actin mRNA species.

### SERCA Activity Measurement

Sarco/endoplasmic reticulum Ca^2+^-ATPase activity was measured based on the inorganic phosphate production using a commercially available kit (Nanjing Jiancheng Bioengineering Institute, Nanjing, China) according to the manufacturer’s instruction. ER fraction was obtained from the kidney using a commercial kit (ER isolation kit, Sigma Aldrich, St. Louis, MO, USA). Briefly, kidney tissues or podocytes were homogenized in isotonic extraction buffer by using a glass homogenizer. After a series of centrifugation (1,000 × *g* for 10 min, 12,000 × *g* for 15 min, and 100,000 × *g* for 1 h), the microsomes were obtained and then homogenized with isotonic extraction buffer. Protein concentration of the ER fraction was quantified and the SERCA activities were determined.

### Podocyte Treatment and Apoptosis Assay

Conditionally immortalized mouse podocytes, kindly provided by Prof. Niansong Wang (Shanghai Sixth People’s Hospital, China) and originally provided by Dr. Peter Mundel (Division of Nephrology, Massachusetts General Hospital, Harvard University), were cultured as previously described (Mundel et al., 1997). Differentiated podocytes were cultured in RPMI 1640 containing 1% FBS for 24 h in six-well plates (apoptosis assay) and 10-cm dishes (protein isolation) before being exposed to different cultural conditions. The cells were allocated into the following groups: (1) normal control (BSA) group incubated in RPMI 1640 containing 1% FBS and BSA, (2) palmitate group incubated in RPMI 1640 with 1% FBS and 250 μM palmitate complexed to BSA (palmitate medium), (3) AS-IV-treated group pretreated with 20, 40 or 80 μM AS-IV for 12 h followed by incubation in palmitate medium for 24 h, and (4) RGZ group pretreated with 10 μM RGZ for 12 h followed by incubation in palmitate medium for 24 h. AS-IV and RGZ were dissolved in DMSO (AMRESCO, Solon, OH, USA) and the final DMSO concentration did not exceed 0.1% (v/v). Palmitate was prepared and podocyte apoptosis was determined by flow cytometry as previously described (Sieber et al., 2010). In brief, a 20 mmol/L solution of palmitic acid (Sigma, St. Louis, MO, USA) in 0.01 mol/l NaOH was complexed to 5% BSA in a molar ratio of 8:1. Then the mixture was sterile filtrated and was added to the serum-containing cell culture medium to achieve a palmitic acid concentration of 250 μM. The final palmitic acid concentration in the medium was measured using a commercial kit (Wako Chemicals, Richmond, VA, USA). BSA was used for control experiments and handled exactly the same as BSA complexed to palmitic acid. Apoptotic podocytes were defined as annexin V-positive/PI-negative (early apoptotic) and annexin V-positive/PI-positive (late apoptotic) cells.

### SERCA2b Knockdown and Overexpression

Oligonucleotide siRNA duplex was synthesized by Invitrogen (Carlsbad, CA, USA). The sequence of mouse SERCA2b siRNA was 5′-GACUCUGCUUUGGAUUAUATT-3′. The sequence of scramble siRNA was 5′-UUCUCCGAACGUGUCACGUTT-3′. Mouse SERCA2b cDNA (GenBank accession no. AJ131821) was synthesized and cloned into pIRES2-EGFP vector (Addgene, Cambridge, MA, USA) to create pIRES2-EGFP-SERCA2b plasmid (protein isolation), and then subcloned into pcDNA3.0 vector (Invitrogen, Carlsbad, CA, USA) to create pcDNA3.0-SERCA2b plasmid (apoptosis assay). The transfection of siRNAs or plasmids in podocytes was carried out with Lipofectamine^®^ RNAiMAX Transfection Reagent or Lipofectamine^®^ 3000 Reagent (both from Invitrogen, Carlsbad, CA, USA), respectively, according to the manufacturer’s instruction. After being transfected with SERCA2b siRNA or pcDNA3.0-SERCA2b/pIRES2-EGFP-SERCA2b plasmid for 24 h, podocytes were pretreated with or without 80 μM AS-IV for 12 h followed by incubation with 250 μM palmitate medium for 24 h and then collected for apoptosis assay or western blotting.

### Intracellular Ca^2+^ Concentration Measurement

Podocytes cultured under indicated conditions were incubated with RPMI 1640 supplemented with 2 μM Fluo-4 AM (Invitrogen, Carlsbad, CA, USA) at 37°C for 30 min. Then the cells were harvested, pelleted, and suspended in ice-cold Ca^2+^-free Krebs-Ringer buffer and assessed by flow cytometry (BD FACSCalibur, Franklin Lakes, NJ, USA). The data were then analyzed with Flowjo 7.6.1 software (FlowJo LLC, Ashland, OR, USA).

### Statistical Analysis

Data are presented as means ± SEM, with *n* representing the number of animals for *in vivo* experiments and independent assays for *in vitro* experiments. Statistical comparisons were performed by using unpaired two-tailed *t*-test or one-way ANOVA followed by the Newman–Keuls multiple comparisons test, with *P-*values < 0.05 being considered significant. Statistical analyses were conducted with Graphpad Prism software 5 software (GraphPad Software, Inc., San Diego, CA, USA). The data and statistical analysis comply with the recommendations on experimental design and analysis in pharmacology (Curtis et al., 2015).

## Results

### AS-IV Improved Metabolic Parameters in *db/db* Mice

**FIGURE 1:**
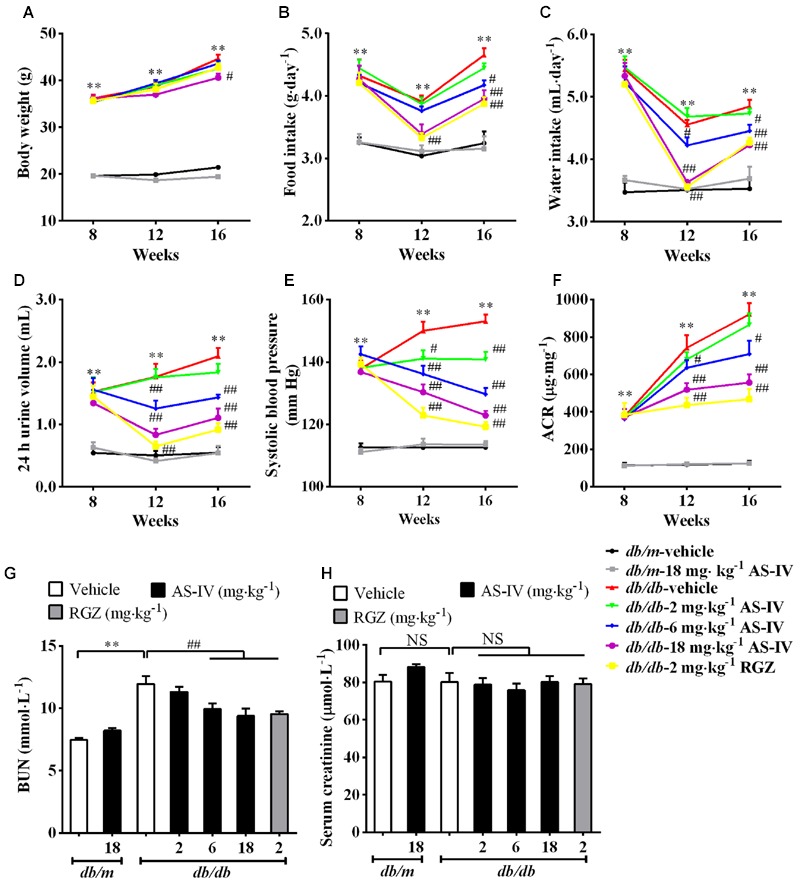
**Effects of AS-IV on metabolic parameters in *db/db* mice.** Eight-week-old *db/db* mice were treated with different doses of AS-IV for eight consecutive weeks. **(A)** Body weight, **(B)** food intake, **(C)** water intake, **(D)** 24 h urine volume, **(E)** systolic blood pressure, and **(F)** ACR were monitored at 4-week intervals. **(G)** Serum BUN and **(H)** serum creatinine were detected at the end of the study. Data are expressed as mean ± SEM. *n* = 10. *^∗∗^P* < 0.01; *^#^P* < 0.05 and *^##^P* < 0.01. NS, no significant difference. One-way ANOVA and Newman–Keuls multiple comparisons test **(A–H)**.

Diabetic *db/db* mice had a significantly higher body weight compared with normal *db/m* mice during the experiment. However, treatment with AS-IV at 18 mg kg^-1^ day^-1^ for 8 weeks significantly reduced body weight in *db/db* mice (**Figure 1A**). Moreover, AS-IV decreased food intake, water intake, 24 h urine volume and hypertension in *db/db* mice (**Figures 1B–E**). *Db/db* mice developed time-dependent, progressive albuminuria (expressed as urinary ACR) and exhibited a significant increase in BUN levels compared with nondiabetic *db/m* mice. Administration of AS-IV at 6 and 18 mg kg^-1^ day^-1^ significantly ameliorated the development of albuminuria (**Figure 1F**) and decreased BUN concentration in *db/db* mice (**Figure 1G**). RGZ had striking beneficial effects on the above parameters without altering body weight. No significant difference in serum creatinine levels was observed among all the groups (**Figure 1H**).

### AS-IV Improved Glucose Homeostasis and Insulin Sensitivity in *db/db* Mice

**FIGURE 2:**
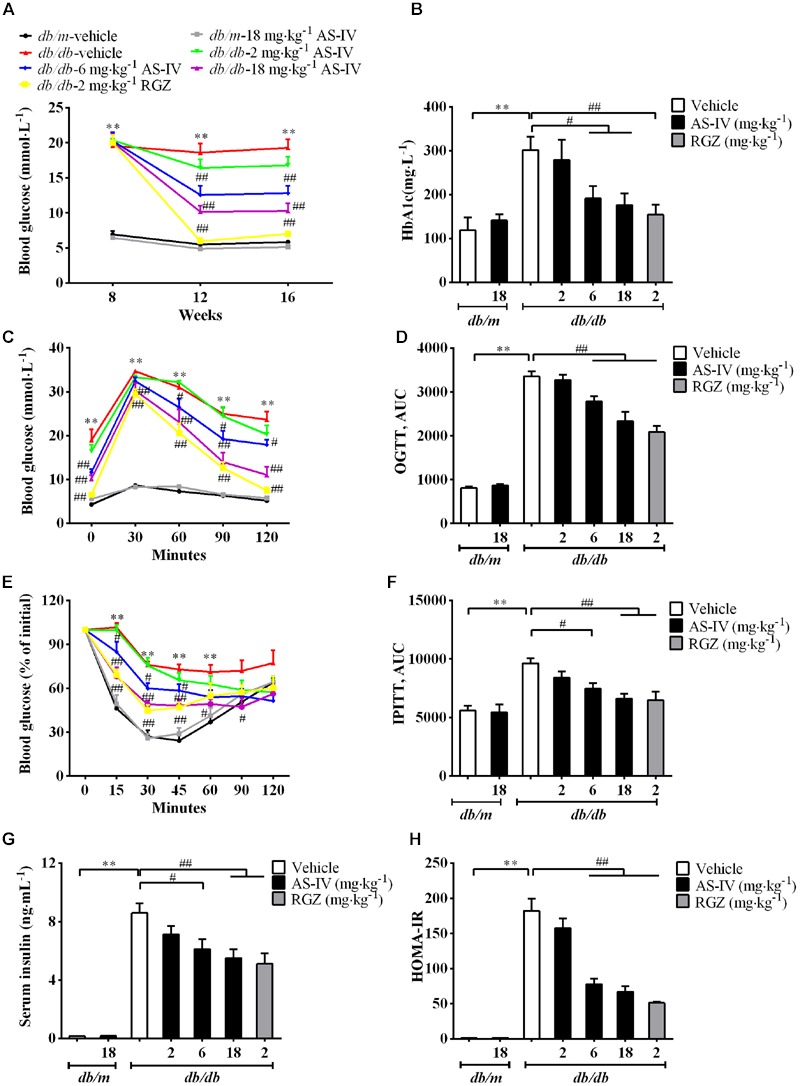
**AS-IV improved glycemic control and insulin levels in *db/db* mice.** Eight-week-old *db/db* mice were treated with different doses of AS-IV for eight consecutive weeks. **(A)** Fasting blood glucose was recorded every 4 weeks. **(B)** serum HbA1c was measured at the end of the study. **(C–F)** Effect of AS-IV treatment on oral glucose tolerance test in *db/db* and *db/m* mice **(C)** expressed as AUC **(D)**, and on intraperitoneal insulin tolerance test in *db/db* and *db/m* mice **(E)** also expressed as AUC **(F)**. **(G)** Serum insulin was measured and **(H)** HOMA-IR was calculated after 8 weeks of intervention. Data are expressed as mean ± SEM. *n* = 10.*^∗∗^P* < 0.01; *^#^P* < 0.05 and *^##^P* < 0.01. One-way ANOVA and Newman–Keuls multiple comparisons test **(A–H)**.

The *db/db* mice developed hyperglycemia as confirmed by significantly elevated fasting blood glucose (**Figure 2A**) and serum HbA1c (**Figure 2B**), which was markedly reduced by 6 and 18 mg kg^-1^ day^-1^ AS-IV (**Figures 2A,B**). OGTT experiment revealed that both 6 and 18 mg kg^-1^ day^-1^ AS-IV-treated *db/db* mice exhibited enhanced glucose clearance as demonstrated by the decreased AUC compared to *db/db-*vehicle group (**Figures 2C,D**). IPITT showed that insulin sensitivity was also improved significantly by 6 and 18 mg kg^-1^ day^-1^ AS-IV, confirmed by the decreases in AUC relative to *db/db*-vehicle group (**Figures 2E,F**). Serum insulin levels and HOMA-IR were also significantly decreased in *db/db*-6 mg kg^-1^⋅day^-1^ AS-IV group and *db/db*-18 mg kg^-1^⋅day^-1^ AS-IV group compared with *db/db*-vehicle group (**Figures 2G,H**). RGZ almost normalized the blood glucose and dramatically improved glucose tolerance and insulin sensitivity in *db/db* mice.

### AS-IV Alleviated Renal Histopathology and Inflammation in *db/db* Mice

**FIGURE 3:**
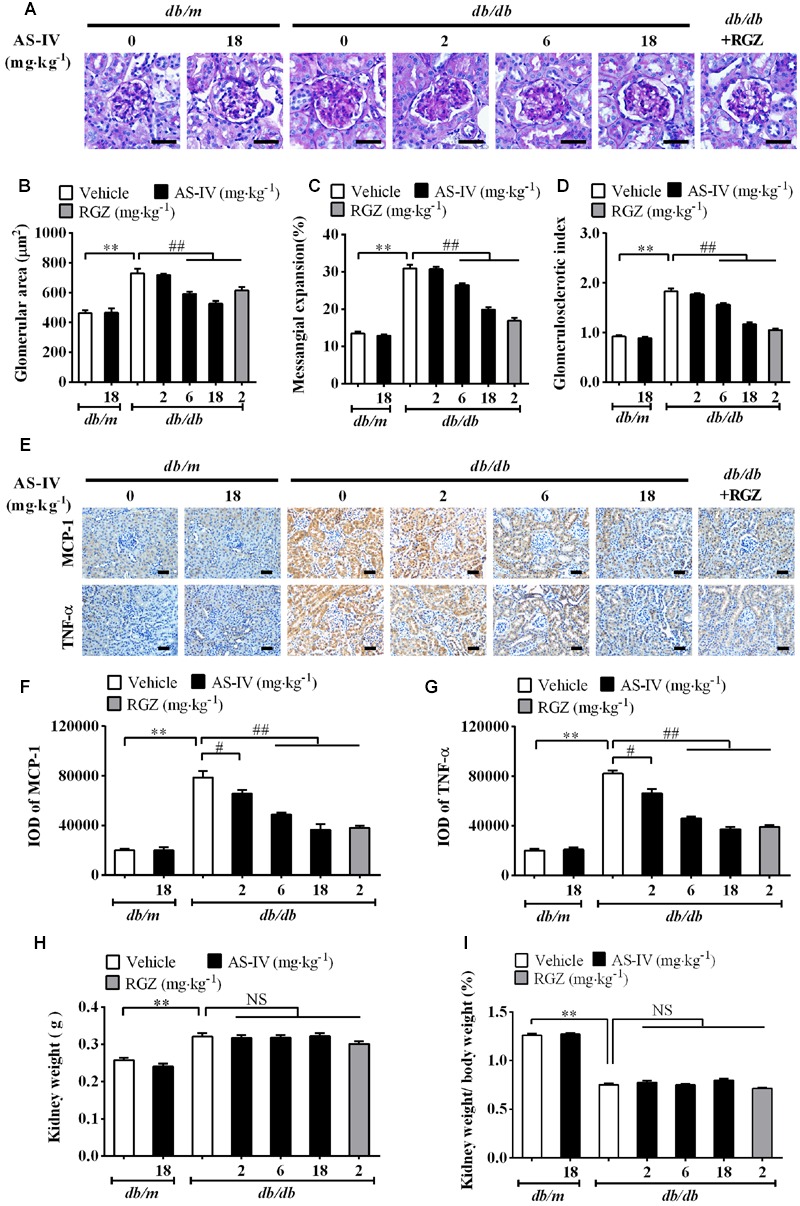
**AS-IV alleviated renal histopathology and renal inflammation in *db/db* mice. (A)** Representative images of PAS staining. Scale bars, 20 μm; Original magnification, ×400. **(B)** Glomerular size in each group. **(C)** Mesangial expansion. **(D)** Glomerulosclerotic score based on PAS staining. **(E)** Representative images of immunohistochemistry staining for MCP-1 and TNF-α. Scale bars, 20 μm; Original magnification, ×400. **(F)** Quantitative result for IOD of MCP-1 in renal cortex areas. **(G)** Quantitative result for IOD of TNF-α in renal cortex areas. **(H)** Kidney weight measurement. **(I)** The percentage of kidney weight/body weight. Data are expressed as mean ± SEM. *n* = 6 **(A–G)** and *n* = 10 **(H,I)**. *^∗∗^P* < 0.01; *^#^P* < 0.05, *^##^P* < 0.01. One-way ANOVA and Newman–Keuls multiple comparisons test **(B–D, F–I)**.

Diabetic nephropathy is a chronic renal complication accompanied by histopathologic changes in kidney (Cooper, 1998). Histological examination of the kidney revealed that the glomerular cross-sectional area was significantly greater in *db/db* mice than that in *db/m* mice at week 16. In addition, *db/db* mice developed more severe mesangial matrix expansion relative to their lean counterparts (**Figure 3A**). The 6 and 18 mg kg^-1^ day^-1^ AS-IV as well as RGZ markedly alleviated glomerular hypertrophy and mesangial matrix expansion (**Figure 3A**). Semiquantitative data further confirmed these observations (**Figures 3B–D**).

Inflammation is involved in DN development (Navarro-Gonzalez and Mora-Fernandez, 2008). The immunohistochemistry staining revealed that MCP-1 and TNF-α expression levels were dramatically elevated in renal tubular epithelial cells of *db/db* mice compared with *db/m* mice. However, AS-IV dose-dependently suppressed the induction of MCP-1 and TNF-α (**Figure 3E**). Quantification of IOD for MCP-1 and TNF-α further confirmed this observation (**Figures 3F,G**). RGZ also displayed powerful activity against renal inflammation. At week 16, kidney weight significantly increased and the percentage of kidney weight/body weight significantly decreased in *db/db* mice relative to nondiabetic *db/m* mice, whereas treatment with AS-IV or RGZ had no obvious effects on these changes (**Figures 3H,I**).

### AS-IV Ameliorated Podocyte Apoptosis in *db/db* Mice

**FIGURE 4:**
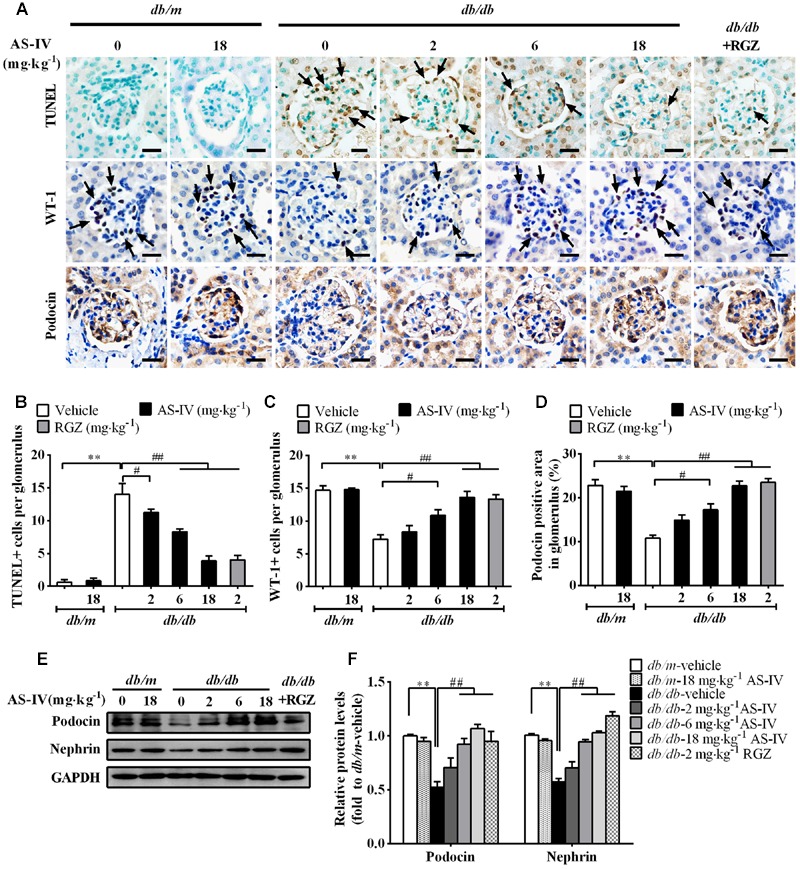
**AS-IV ameliorated podocyte apoptosis in *db/db* mice. (A)** TUNEL staining and immunohistochemistry staining for WT-1 and Podocin. Scale bars, 10 μm; Original magnification, ×400. **(B)** Quantification of TUNEL-positive cells in each group. Data was expressed as TUNEL-positive cell number per glomerulus. **(C)** Quantification of podocyte number in each group. Results were represented as WT-1-positive podocyte number per glomerulus. **(D)** Quantification of the Podocin-positive area in glomeruli. **(E,F)** Representative immunoblots **(E)** and densitometric quantification **(F)** of Podocin and Nephrin expression in total lysates of kidney cortex from each group. Data are expressed as mean ± SEM. *n* = 6.*^∗∗^P* < 0.01; *^#^P* < 0.05 and *^##^P* < 0.01. One-way ANOVA and Newman–Keuls multiple comparisons test **(B–D,F)**.

Podocyte apoptosis/loss is closely associated with the pathogenesis of albuminuria and glomerulosclerosis, thus being considered as a predictor for the progression of DN (Reidy et al., 2014). The number of apoptotic cells was dramatically increased in *db/db* mice relative to *db/m* mice as demonstrated by TUNEL staining, while AS-IV dose-dependently attenuated apoptotic cell number (**Figures 4A,B**). Immunohistochemistry staining of WT-1 (podocyte nuclei) and Podocin (podocyte foot processes) revealed marked podocyte loss in *db/db* mice, while AS-IV effectively prevented the loss of podocytes (**Figure 4A**), which was confirmed by semiquantitation of WT-1-positive cells and podocin-positive area in the glomeruli (**Figures 4C,D**). Western blot demonstrated that the expression levels of Podocin and Nephrin were reduced in renal cortex of *db/db* mice but was restored by AS-IV in a dose-dependent manner (**Figures 4E,F**). RGZ treatment significantly alleviated podocyte apoptosis in *db/db* mice (**Figure 4**).

### AS-IV Restored the Expression and Activity of SERCA2 in the Kidney Cortex of *db/db* Mice

**FIGURE 5:**
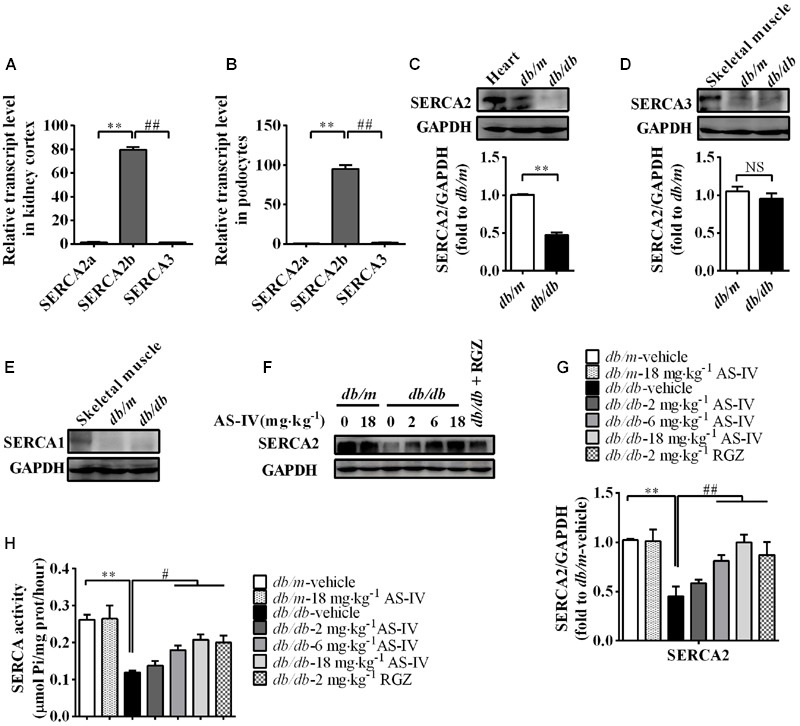
**AS-IV treatment restored SERCA2 expression and activity in the kidney of *db/db* mice. (A,B)** qRT-PCR quantitation of SERCA2a, SERCA2b, and SERAC3 in *db/m* mouse kidney cortex **(A)** and cultured mouse podocyte cell line **(B)**. **(C–E)** Western blot analyses and densitometric quantification of SERCA2 **(C)**, SERCA3 **(D)** and SERCA1 **(E)** levels in the lysates of kidney cortex from *db/m* and *db/db* mice. Heart and skeletal muscle lysate of *db/m* mice was used as positive control. **(F)** The effect of AS-IV on SERCA2 expression in *db/db* mice determined by western blot. **(G)** Densitometric quantification of SERCA2 expression in the kidney cortex lysates from each group. **(H)** Relative SERCA2 activity in ER extraction of the kidney from each group. *^∗∗^P* < 0.01; *^##^P* < 0.01. NS, no significant difference. Data are expressed as mean ± SEM. *n* = 6. Student’s *t*-test **(A–D)**, one-way ANOVA and Newman–Keuls multiple comparisons test **(G,H)**.

To gain molecular mechanistic insights into the protective action of AS-IV against DN, alterations in SERCA expression were measured. qRT-PCR by using equally efficient primers adopted by Kono et al. showed that SERCA2b was the most prevalent mRNA species within the kidney and conditionally immortalized mouse podocyte cell line, while both SERCA2a and SERCA3 were expressed at extremely low levels (**Figures 5A,B**). In addition, western blot results showed that SERCA2 protein levels were significantly reduced by 50% in the renal cortex of *db/db* mice relative to that of *db/m* controls and there was no significant difference in SERCA3 protein levels between *db/m* and *db/db* mice (**Figures 5C,D**). SERCA1 expression could not be detected (**Figure 5E**). Given the qRT-PCR and western blot data, our following experiment focused on SERCA2b isoform. The decreased SERCA2 expression in *db/db* mouse kidney was restored by AS-IV in a dose-dependent manner (**Figures 5F,G**). In parallel to a marked depression in SERCA2 expression, SERCA activity was remarkably decreased by 50% in *db/db* mouse kidney compared to *db/m* mice. However, treatment with AS-IV at 6 and 18 mg kg^-1^ day^-1^ remarkably rescued SERCA activity in the kidneys of *db/db* mice (**Figure 5H**). RGZ significantly prevented the reductions in SERCA2 expression and activity (**Figures 5F–H**).

### AS-IV Inhibited ER Stress and ER Stress-Induced Apoptotic Pathway in the Kidney of *db/db* Mice

**FIGURE 6:**
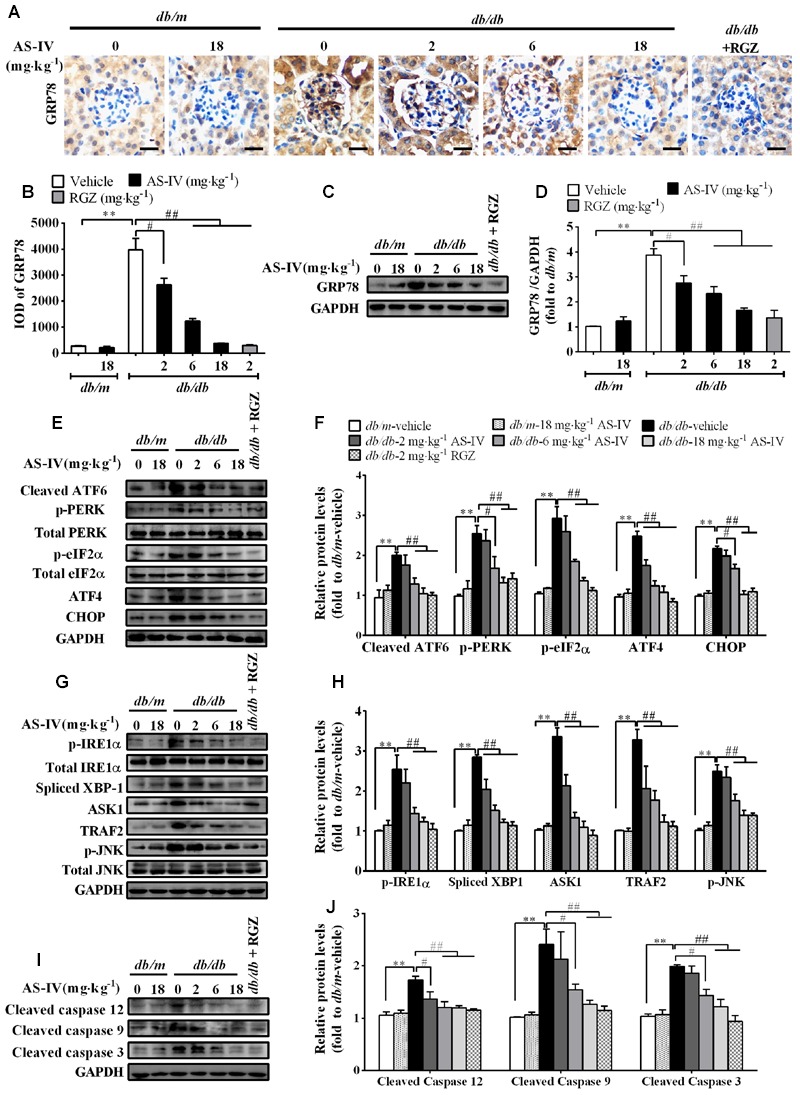
**AS-IV attenuated ER stress and ER stress-induced apoptotic pathway in the kidney of *db/db* mice. (A,B)** Representative pictures for GRP78 immunohistochemistry staining **(A)** and quantification of IOD **(B)** for GRP78 in each group. Scale bars, 10 μm; Original magnification, ×400. **(C,D)** Western blot analyses **(C)** and densitometric quantification **(D)** of GRP78 expression in the kidney cortex lysates from each group. **(E,F)** Representative immunoblots **(E)** and densitometric quantification **(F)** of ATF6, p-PERK, p-eIF2α, ATF4, CHOP expression in total lysates of kidney cortex from each group. **(G,H)** Representative immunoblots **(G)** and densitometric quantification **(H)** of p-IRE1α, spliced-XBP1, TRAF2, ASK1, and phospho-JNK^Thr183/Tyr185^ expression in total lysates of kidney cortex from each group. **(I,J)** Representative immunoblots **(I)** and densitometric quantification **(J)** of cleaved caspase 12, cleaved caspase 9 and cleaved caspase 3 expression in total lysates of kidney cortex from each group. Data are expressed as mean ± SEM. *n* = 6. *^∗∗^P* < 0.01; *^#^P* < 0.05 and *^##^P* < 0.01. One-way ANOVA and Newman–Keuls multiple comparisons test **(B,D,F,H,J)**.

Aberrant expression or activity of SERCA2 leads to ER Ca^2+^ dysregulation and triggers ER stress (Lytton et al., 1991; Park et al., 2010; Fu et al., 2011). Immunohistochemistry staining revealed that the expression of ER chaperone GRP78 was dramatically up-regulated in the renal cortex of *db/db* mice compared with *db/m* mice, which indicated the activation of ER stress, but attenuated by AS-IV in a dose-dependent manner (**Figures 6A,B**). Western blot analysis further confirmed this observation (**Figures 6C,D**). Three ER stress master regulators ATF6, PERK, IREα and their downstream targets, such as eIF2α, ATF4, and XBP1 were activated, indicating the activation of all three branches of UPR signaling pathway. However, their activations were significantly suppressed by 6 and 18 mg kg^-1^ day^-1^ AS-IV treatment (**Figures 6E–J**). Three known mediators of ER stress-induced apoptosis, including ATF6/PERK downstream molecule CHOP, IRE1α downstream molecule phospho-JNK, and cleaved caspase-12, were all enhanced in *db/db* mice (**Figures 6E–J**). However, their expression was remarkably reduced by AS-IV, strongly manifesting that AS-IV abrogates ER stress and ER stress-induced apoptosis in *db/db* mice.

### AS-IV Suppressed Mitochondria-Mediated Apoptotic Pathway in the Kidney of *db/db* Mice

**FIGURE 7:**
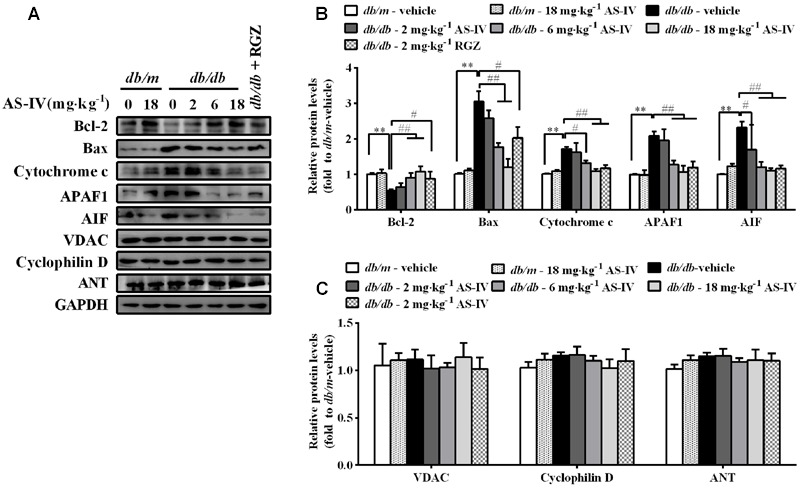
**Effects of AS-IV on mitochondrial-mediated apoptotic pathway in the kidney of *db/db* mice. (A)** Representative immunoblots showing Bcl-2, Bax, cytochrome *c*, APAF1, AIF and the permeability transition pore (PTP) protein levels in renal cortex lysates obtained from each group. **(B)** Quantification of Bcl-2, Bax, cytochrome *c*, APAF1, AIF expression showed in panel A. **(C)** Quantification of PTP protein expression showed in panel A. Data are expressed as mean ± SEM. *n* = 6. *^∗∗^P* < 0.01; *^#^P* < 0.05, *^##^P* < 0.01. One-way ANOVA and Newman–Keuls multiple comparisons test **(B,C)**.

In parallel, disturbance of ER Ca^2+^ homeostasis by ER stress also evokes mitochondria-mediated apoptosis. Western blot analysis showed a decrease in anti-apoptotic protein Bcl-2 expression and an increase in the protein levels of pro-apoptotic proteins, such as Bax, cytochrome *c*, APAF1, and AIF in renal cortex of *db/db* mice compared with *db/m* mice, while AS-IV restored their expression (**Figures 7A,B**). There was no significant difference in the expression levels of VDAC, ANT, and cyclophilin D, components of the permeability transition pore (PTP), between *db/m* mice and *db/db* mice (**Figures 7A,C**).

### AS-IV Restored SERCA2 Expression and Inhibited ER Stress-Induced Apoptosis in Palmitate-Stimulated Podocytes

**FIGURE 8:**
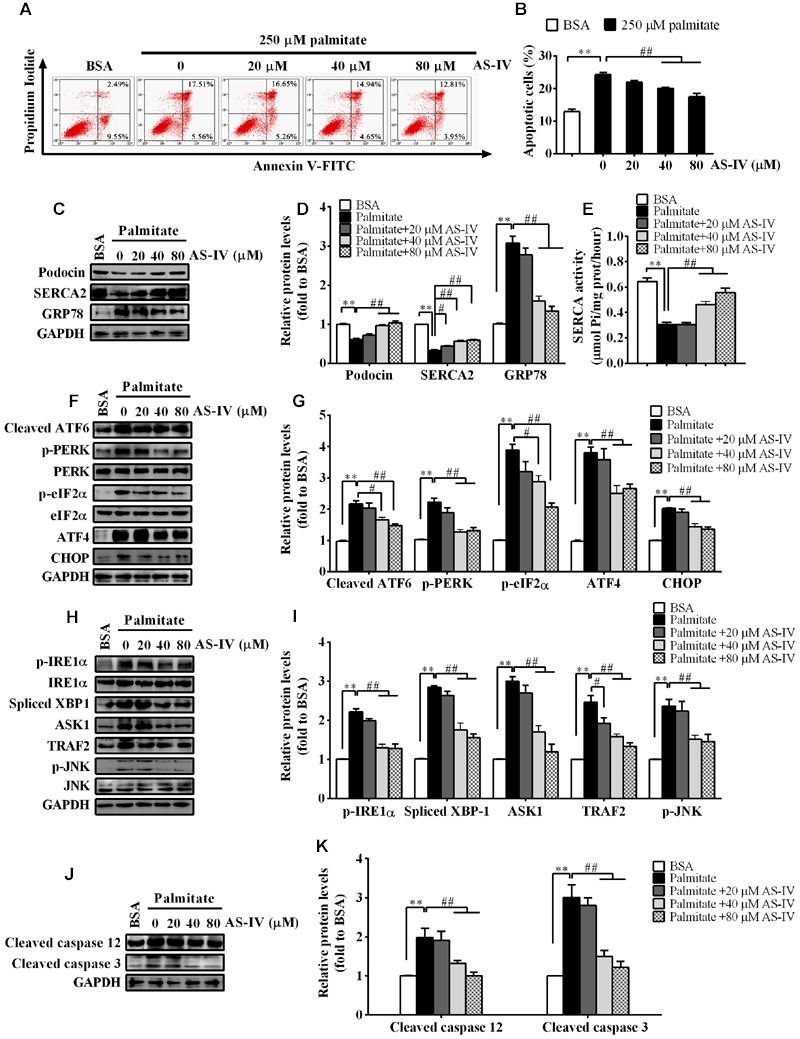
**AS-IV restored SERCA2 expression and inhibited ER stress-mediated apoptosis in palmitate-stimulated podocytes.** Podocytes were pretreated with or without AS-IV at the indicated concentrations (20, 40, and 80 μM respectively) for 12 h followed by 250 μM palmitate exposure for 24h. **(A)** Representative flow cytometry results for podocytes under different cultural conditions. **(B)** Semiquantitative data showing percentage of apoptotic podocytes under different cultural conditions. **(C,D)** Representative immunoblots **(C)** and quantification **(D)** of Podocin, SERCA2 and GRP78 under different conditions. **(E)** SERCA activity under different conditions. **(F,G)** Representative immunoblots **(F)** and quantification **(G)** of key molecules in ATF6-CHOP/PERK–eIF-2α–ATF4–CHOP signaling pathways under different conditions. **(H,I)** Representative immunoblots **(H)** and quantification **(I)** of key molecules in IRE1–XBP1/IRE1-TRAF2–ASK1–JNK signaling pathway. **(J,K)** Representative immunoblots **(J)** and quantification **(K)** of cleaved caspase 12 and cleaved caspase 3 under different conditions. Data are expressed as mean ± SEM. *n* = 3–5. *^∗∗^P* < 0.01; *^#^P* < 0.05 and *^##^P* < 0.01. One-way ANOVA and Newman–Keuls multiple comparisons test **(B,D,E,G,I,K)**.

*db/db* mouse is an obese mouse model for type 2 diabetes, characterized by dyslipidemia and an increase in plasma levels of long-chain free fatty acids (FFAs) (Wahba and Mak, 2007; Mooradian, 2009). Saturated FFAs such as palmitate are pro-apoptotic factors and lead to podocyte apoptosis by inducing ER stress (Martinez et al., 2008; Sieber et al., 2010). We further confirmed the protective effect of AS-IV on ER stress-induced apoptosis using palmitate-induced podocyte injury model *in vitro*. Palmitate induced a significant podocyte apoptosis while treatment with AS-IV significantly decreased palmitate-induced podocyte apoptosis in a concentration-dependent manner, manifested by flow cytometric analysis (**Figures 8A,B**). Podocin expression was significantly downregulated in palmitate-stimulated podocytes, accompanied with a dramatic reduction in SERCA2 expression and activity and an increase in GRP78 expression. However, AS-IV markedly restored Podocin expression as well as SERCA2 expression and activity, and GPR78 expression was significantly attenuated at the same time (**Figures 8C–E**). Further study showed that AS-IV suppressed the activation of three UPR arms including ATF6, PERK-eIF-2α-ATF4, and IRE1α-XBP1 (**Figures 8F–I**). ER stress-mediated apoptotic pathway was activated by palmitate but inhibited by AS-IV, as manifested by the down-regulation of CHOP, TRAF2, ASK1, phospho-JNK and cleaved caspase 12 in AS-IV treated group relative to those in palmitate group (**Figures 8F–K**).

### AS-IV Restored Ca^2+^ Homeostasis and Suppressed Mitochondria-Mediated Apoptotic Pathway in Palmitate-Stimulated Podocytes

**FIGURE 9:**
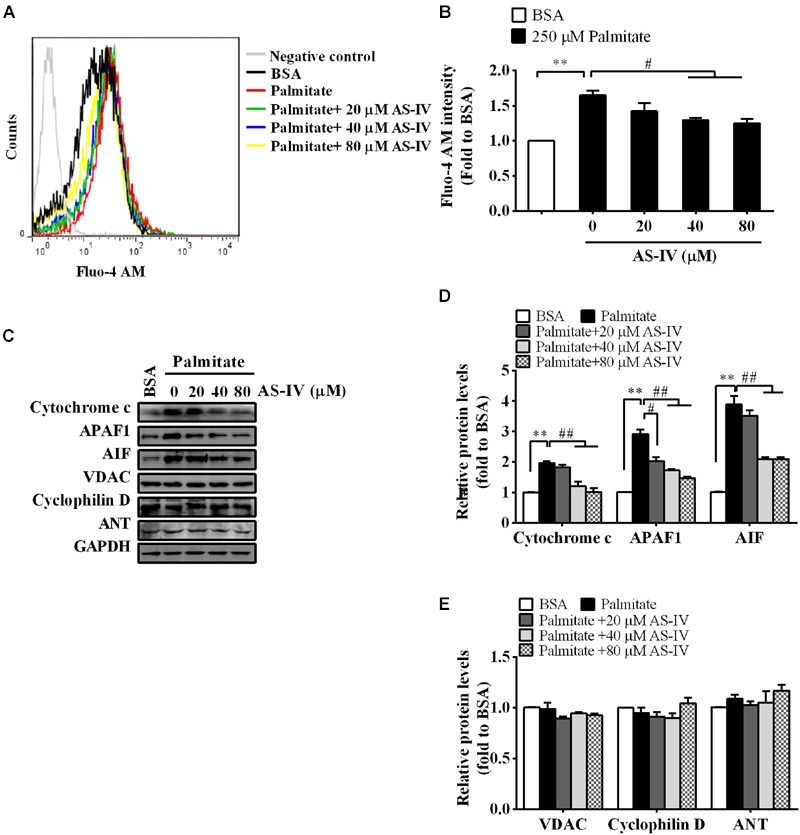
**AS-IV normalized intracellular Ca^2+^ levels and suppressed mitochondria-mediated apoptotic pathway in palmitate-stimulated podocytes.** Podocytes were pretreated with or without AS-IV at the indicated concentrations (20, 40, and 80 μM, respectively) for 12 h followed by 250 μM palmitate incubation for 24 h. **(A)** Representative pictures of Fluo-4 AM staining showing intracellular Ca^2+^ levels under different conditions. **(B)** The relative fluo-4 AM fluorescence intensity under different conditions. **(C)** Representative immunoblots for cytochrome *c*, APAF1, AIF, and the PTP protein levels in podocytes under different conditions. **(D)** Quantification of cytochrome *c*, APAF1, AIF expression in podocytes under different conditions. **(E)** Quantification of the PTP protein expression levels in podocytes under different conditions. Data are expressed as mean ± SEM. *n* = 3–5. *^∗∗^P* < 0.01; *^#^P* < 0.05 and *^##^P* < 0.01. One-way ANOVA and Newman–Keuls multiple comparisons test **(B,D,E)**.

Consistent with a decrease in SERCA2 expression and activity, palmitate resulted in a rise in basal cytosolic Ca^2+^ levels. AS-IV restored basal cytosolic Ca^2+^ levels in a dose-dependent manner (**Figures 9A,B**). Meanwhile, palmitate induced the expression of cytochrome *c*, APAF1 and AIF in podocytes, suggesting the activation of mitochondria-mediated apoptotic pathway. However, the induction of cytochrome *c*, APAF1, and AIF was markedly suppressed by AS-IV in a dose-dependent manner. The expression levels of PTP proteins, including VDAC, Cyclophilin D, and ANT, were not significantly changed under different cultural conditions (**Figures 9C–E**).

### AS-IV Attenuated Palmitate-Induced Podocyte Apoptosis by Regulating SERCA2b Expression

**FIGURE 10:**
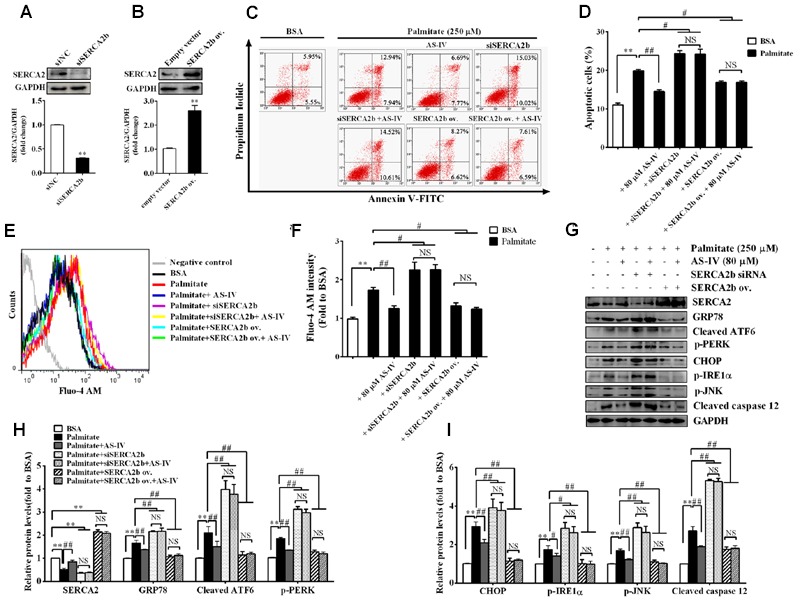
**SERCA2 mediated the inhibitory effect of AS-IV on palmitate-induced podocyte apoptosis.** Podocytes were transfected with scramble siRNA (siNC), specific SERCA2b siRNA (siSERCA2b), empty vector and SERCA2b overexpression (ov.) plasmid, respectively, for 24 h, and then pretreated with or without 80 μM AS-IV for 12 h followed by stimulation with 250 μM palmitate for 24 h. **(A)** Western blot and densitometric quantification of the endogenous SERCA2b expression in podocytes transfected with siSERCA2b or siNC. **(B)** Western blot and densitometric quantification of the total SERCA2b expression in podocytes transfected with SERCA2b ov. plasmid or empty vector. **(C,D)** Representative flow cytometry results **(C)** and semiquantitative data **(D)** for podocyte apoptosis under different cultural conditions. **(E)** Representative picture of Fluo-4 AM staining showing intracellular Ca^2+^ levels under different conditions. **(F)** The relative fluo-4 AM fluorescence intensity depicting intracellular Ca^2+^ levels under different conditions. **(G–I)** Representative immunoblots **(G)** and quantification **(H,I)** of SERCA2 and ER stress-associated proteins under different conditions. Data are expressed as mean ± SEM. *n* = 3–5. *^∗∗^P* < 0.01; *^#^P* < 0.05 and *^##^P* < 0.01. NS, no significant difference. Student’s *t*-test **(A,B)**, one-way ANOVA and Newman–Keuls multiple comparisons test **(D,F,H,I)**.

To further confirm the role of SERCA2b in mediating the effect of AS-IV on ER stress-induced podocyte apoptosis, we knocked down endogenous SERCA2b expression using a SERCA2b-specific siRNA or overexpressed the exogenous SERCA2b by transfection with SERCA2b overexpression plasmid. SERCA2 expression was reduced by 70% after SERCA2b siRNA transfection (**Figure 10A**) and increased significantly after SERCA2b overexpression plasmid transfection (**Figure 10B**). We then transfected SERCA2b siRNA or SERCA2 overexpression plasmid into podocytes to investigate the role of SERCA2b in the action of AS-IV. Flow cytometric analysis showed that SERCA2b knockdown resulted in a significant increase in palmitate-induced apoptosis and abolished the protective effect of AS-IV on podocytes, while SERCA2b overexpression significantly decreased palmitate-induced apoptosis and achieved a similar beneficial effect as AS-IV on podocytes (**Figures 10C,D**). Further data showed that SERCA2b knockdown markedly increased palmitate-induced intracellular Ca^2+^ rise and ER stress, enhanced the activation of ER stress-mediated apoptotic pathway and blocked the inhibitory effect of AS-IV on palmitate-induced intracellular Ca^2+^ alteration and ER stress, while SERCA2b overexpression significantly attenuated palmitate-induced intracellular Ca^2+^ rise and ER stress, repressed the activation of ER stress-mediated apoptotic pathway, and achieved a similar potential as AS-IV on intracellular Ca^2+^ dysregulation and ER stress (**Figures 10E–I**).

## Discussion

In the present study, with *db/db* mice, a model of spontaneous DN from type 2 diabetes, we confirmed the pronounced protective effect of AS-IV against DN. Importantly, we found a significant reduction of SERCA2 expression and activity in *db/db* mouse kidney and cultured mouse podocytes exposed to palmitate, accompanied by an increase of cytosolic Ca^2+^ levels, an induction of ER stress and the activation of three UPR pathways, ER stress-mediated apoptotic pathway and mitochondria-mediated apoptotic pathway, which lead to podocyte apoptosis *in vivo* and *in vitro.* However, AS-IV restored SERCA2 expression, rescued intracellular Ca^2+^ homeostasis, and reversed the activation of UPR and ER stress-mediated apoptotic pathway as well as mitochondria-mediated apoptotic pathway, concomitant with a marked attenuation of podocyte apoptosis *in vivo* and *in vitro.* Further study revealed that SERCA2b knockdown abolished the inhibitory effect of AS-IV on ER stress-induced apoptotic pathway, while SERCA2b overexpression exhibited an anti-apoptotic action. Our data suggest that the beneficial effect of AS-IV on DN is associated with the alleviation of ER stress via upregulating SERCA2 expression, which subsequently alleviates ER stress-induced podocyte apoptosis, thereby preventing the development of DN.

The SERCA pump is responsible for maintaining ER Ca^2+^ homeostasis and serves as an essential gatekeeper for ER function (Vangheluwe et al., 2005). Recently, SERCA dysfunction has been linked with experimental and human pathology, including heart failure (Shareef et al., 2014), diabetes (Zarain-Herzberg et al., 2014), vascular diseases (Adachi et al., 2004; Davies, 2015), tumor (Wang et al., 2011), as well as muscular dystrophy (Gehrig et al., 2012; Schneider et al., 2013). Of all the SERCA isoforms, SERCA2b is ubiquitously expressed and considered the housekeeping isoform, while SERCA2a is a muscle-specific isoform expressed in type I (slow) skeletal, cardiac, and smooth muscle (Vangheluwe et al., 2005). SERCA1 is restricted to fast-twitch skeletal muscle and SERCA3 is present in a limited number of nonmuscle cells including platelets and endothelial cells (Vangheluwe et al., 2005). Here, we identified SERCA2b as the most highly expressed isoform at transcriptional level in both mouse kidney cortex and conditionally immortalized mouse podocyte cell line, whereas SERCA2a and SERCA3 were expressed at very low levels (**Figure 5**). We detected no expression of SERCA1. Therefore, our following study focused on SERCA2b isoform.

The link between SERCA dysfunction and ER stress has been well established. Thapsigargin, an inhibitor of SERCA, abolishes Ca^2+^ uptake into the ER, leading to ER stress and subsequent initiation of UPR (Lytton et al., 1991). Several groups have revealed that SERCA2 activity and/or expression is reduced in metabolic syndrome, and the reduction results in ER stress and ER stress-induced apoptosis. The restoration of SERCA2 activity or expression can ameliorate ER stress and in turn improve metabolic abnormalities (Park et al., 2010; Kang et al., 2015). SERCA2b in diabetic islet is downregulated, resulting in Ca^2+^ dysregulation, insulin-secretory defects and ER stress, while restoration of islet SERCA2 levels by pioglitazone, the agonist of PPARγ, reduces ER stress and enhances islet function (Evans-Molina et al., 2009; Kono et al., 2012). SERCA2b protein and mRNA levels are dramatically reduced in the liver of *ob/ob* mice accompanied by the increased ER stress and apoptosis, and overexpressed SERCA2b or pharmacologically activated SERCA2b in the liver of obese and diabetic mice improves glucose homeostasis, reduces steatohepatitis, and inhibits ER stress and ER stress-induced apoptosis via ameliorating PERK/eIF2α/CHOP and IRE1α/JNK/XBP1 pathways (Park et al., 2010; Kang et al., 2015). In hearts of OLETF, a rat model of type 2 diabetes, SERCA2a protein is reduced and this reduction was restored by treatment with 4-phenyl butyric acid (PBA), an ER stress inhibitor (Takada et al., 2012). Impaired SERCA activity contributes to cardiomyocyte dysfunction in insulin resistant rats (Wold et al., 2005). All the above reports highlight a potential pathological role for SERCA2 in the development of metabolic syndrome. In the present study, we found that SERCA2 expression and SERCA activity were significantly decreased in the kidney cortex of *db/db* mice (**Figure 5**) and mouse podocytes incubated with palmitate (**Figure 8**), paralleled by an elevation of intracellular Ca^2+^ levels in palmitate-incubated podocytes (**Figure 9**), an induction of ER stress and the activation of three UPR arms as well as ER stress-mediated apoptotic pathway as evidenced by increased expression of GRP78, cleaved ATF6, phospho-PERK, phospho-eIF2α, ATF4, CHOP, phospho-IRE1α, spliced XBP1, ASK1, TRAF2, phospho-JNK, and cleaved caspase 12 (**Figures 6** and **8**). However, the increases in cytosolic Ca^2+^ levels and the induction of ER stress indicators were inhibited by AS-IV in a dose-dependent manner along with the restoration of SERCA2 expression and SERCA activity *in vivo* and *in vitro* (**Figures 5, 8**, and **9**). Further study revealed that knockdown of SERCA2 enhanced palmitate-induced intracellular Ca^2+^ rise, aggravated palmitate-induced ER stress and blunted SERCA2b up-regulation by AS-IV, while SERCA2 overexpression diminished palmitate-induced intracellular Ca^2+^ rise, alleviated ER stress and prevented the initiation of UPR with a similar potency achieved by AS-IV. Meanwhile, SERCA2b knockdown blocked the inhibitory effect of AS-IV on intracellular Ca^2+^ dysregulation, ER stress and ER stress-mediated apoptotic pathway while SERCA2 overexpression exhibited an opposite effect (**Figure 10**). These data strongly provide evidence that AS-IV suppresses ER stress at least in part through upregulating SERCA2b expression. In parallel with the upregulation of CHOP, phospho-JNK and cleaved caspase 12, positive indicators of ER stress-mediated apoptotic pathway, podocyte apoptosis was significantly induced but markedly attenuated by AS-IV *in vivo* and *in vitro* (**Figures 4** and **8**). The findings are in agreement with recent reports that ER stress plays an important role in podocyte apoptosis (Sieber et al., 2010; Cao et al., 2016) and AS-IV has pharmacological activities against podocyte injury through ER stress inhibition (Chen Y. et al., 2014; Wang et al., 2015).

Endoplasmic reticulum and mitochondria are physiologically and functionally interacted, with calcium signaling being the hub of the interaction between the two organelles. Recent studies demonstrated that disruption of ER Ca^2+^ homeostasis contributes to apoptosis through mitochondria-mediated apoptotic pathway. Enhanced mitochondria uptake of Ca^2+^ released from the ER leads to the release of pro-apoptotic factors such as cytochrome *c*, smac/Diablo, and AIF. Cytochrome *c* then binds to APAF1, leading to the cleavage of procaspase 9 into active caspase 9 and subsequent activation of caspase 3 (Malhotra and Kaufman, 2011). In our study, mitochondria-mediated apoptotic pathway was evoked in *db/db* mice and palmitate-stimulated podocytes, confirmed by elevated expression of cytochrome *c*, APAF1 and AIF, but the induction of these proteins was significantly attenuated by AS-IV (**Figures 7** and **9**). In aggregate, AS-IV attenuated ER stress-induced podocyte apoptosis probably through simultaneous inhibition of both ER stress-mediated apoptotic pathway and mitochondria-mediated apoptotic pathway.

Remarkably, the renoprotective effect of AS-IV-dependent SERCA2 restoration was confirmed in *db/db* mice. Treatment with AS-IV at 18 mg kg^-1^ day^-1^ for 8 weeks significantly reduced body weight gain associated with the decreases in food consumption, water intake and 24 h urine volume in *db/db* mice during the whole experiment (**Figure 1**). Besides, AS-IV improved renal function of *db/db* mice as manifested by reduced albuminuria and serum BUN levels (**Figure 1**). Serum creatinine levels were not changed significantly, probably due to that 16-week-old *db/db* mice are in the early stage of DN and serum creatinine concentration has not increased significantly yet (Kaizu and Komine, 2001). SERCA2 restoration by AS-IV improved glucose homeostasis in *db/db* mice, as confirmed by the lowered blood glucose and the improved glucose tolerance and insulin sensitivity (**Figure 2**). This is indeed consistent with the previous reports that SERCA2b overexpression or SERCA2b activation can improve glucose homeostasis in diabetic *ob/ob* mice (Park et al., 2010; Kang et al., 2015) and that cardiac-specific SERCA overexpression improves whole body glucose homeostasis by rescuing diabetes induced-alterations in cardiac glucose transport (Waller et al., 2015). Moreover, AS-IV attenuated glomerular hypertrophy, mesangial matrix expansion, and renal inflammation in a dose-dependent manner in *db/db* mice (**Figure 3**). All these observations demonstrate a pronounced renoprotective effect of AS-IV against DN. Though it is unclear whether the improved renal function is the direct effect of AS-IV on podocytes or the indirect effect of improved metabolic milieu of type 2 diabetes such as improved glycemic control, or a combination of both effects, the *in vitro* studies do at least help define that AS-IV had direct protective action on podocytes under diabetic conditions. It is noteworthy that lean *db/m* mice treated with AS-IV did not exhibited any obvious alterations in either metabolic homeostasis or histology, indicating that AS-IV is unlikely to induce abnormalities in metabolically healthy animals.

Thiazolidinedione drugs, such as RGZ or pioglitazone, agonists of PPARγ, are used to treat type 2 diabetes by improving insulin sensitivity (Campbell, 2005). There are accumulating evidence of diverse effects of PPARγ agonists on podocytes and DN progression (Benigni et al., 2006; Kanjanabuch et al., 2007). In our study, RGZ significantly normalized metabolic and biochemical parameters and preserved renal function in *db/db* mice. Furthermore, PPARγ expression was downregulated in renal cortex of diabetic *db/db* mice and palmitate-incubated podocytes. Both AS-IV and RGZ could restored PPARγ expression (Supplementary Figures 1 and 2C,D), which was consistent with the recent finding that AS-IV is a novel natural PPARγ agonist (Wang et al., 2016). PPARγ was identified as a direct transcriptional regulator of the SERCA2 gene in β-cells under diabetic conditions (Kono et al., 2012). Similarly, we found that AS-IV and RGZ improved SERCA2 expression in palmitate-incubated podocytes (Supplementary Figures 2C,D), which raises the attractive possibility that SERCA2 is a direct downstream target for PPARγ since SERCA genes contain conserved PPAR-responsive elements within their regulatory regions (Zarain-Herzberg and Alvarez-Fernandez, 2002). In addition, RGZ rescued intracellular Ca^2+^ homeostasis, mitigated ER stress and exhibited cytoprotection against palmitate-induced podocyte apoptosis (Supplementary Figures 2 and 3). Thus, we propose that AS-IV may act as a PPARγ agonist and mitigate ER stress in podocytes by a mechanism involving at least SERCA activity, but further studies are still needed to clarify the mechanism.

## Conclusion

Our current study has suggested for the first time that the improvement of SERCA2 expression and function by AS-IV prevents the progression of DN in *db/db* mice. The restoration of SERCA2 expression is associated with concomitant restoration of intracellular Ca^2+^ homeostasis and enhancement of ER function, which attenuate ER stress and subsequently relieve ER stress-induced podocyte apoptosis, finally inhibiting the progression of DN. We provide evidence that AS-IV may be a novel therapeutic agent modulating SERCA2b expression to prevent the development of DN.

## Ethics Statement

All the work was carried out in accordance with the approved guidelines for the use of experimental animals in Putuo Hospital, Shanghai University of Traditional Chinese Medicine.

## Author Contributions

HG and AC designed the study, performed part of experiments, interpreted the data and performed data analysis. SC, YiW, YZ, XM, and CL performed part of experiments. HW, YuW, XZ, and WP interpreted the data, drafted the manuscript and revised it critically for intellectual content. All authors read and approved the final version of the manuscript before submission.

## Conflict of Interest Statement

The authors declare that the research was conducted in the absence of any commercial or financial relationships that could be construed as a potential conflict of interest.

## Abbreviations

AIF: apoptosis-inducing factor
ANT: adenine nucleotide translocase
APAF1: apoptosis protease-activating factor 1
AS-IV: astragaloside IV
ASK1: apoptosis signal-regulating kinase 1
ATF: activating transcription factor
BUN: blood urea nitrogen
CHOP: C/EBP homology protein
DN: diabetic nephropathy
eIF2α: eukaryotic translation initiation factor 2α
ER: endoplasmic reticulum
GRP78: glucose-regulated protein 78
HOMA-IR: homeostatic model assessment of insulin resistance
IOD: integrated optical density
IRE1: inositol-requiring enzyme 1
PAS: periodic acid-Schiff
PERK: protein kinase R-like ER kinase
PPARγ: peroxisome proliferator-activated receptor γ
RGZ: rosiglitazone
SERCA: sarco/endoplasmic reticulum Ca^2+^-ATPase
siRNA: small interference RNA
UPR: unfolded protein response
XBP1: X-box binding protein 1
