# Supplementary material for: Astragaloside IV Attenuates Podocyte Apoptosis Mediated by Endoplasmic Reticulum Stress through Upregulating Sarco/Endoplasmic Reticulum Ca2+-ATPase 2 Expression in Diabetic Nephropathy
Source: Front Pharmacol. 2016 Dec 21;7:500. doi: 10.3389/fphar.2016.00500 (PMC5174081; doi:10.3389/fphar.2016.00500)
Supplement: Supplementary file 1 [file Data_Sheet_1.PDF]

Supplementary Material

Astragaloside IV attenuates podocyte apoptosis mediated by endoplasmic reticulum stress through upregulating Sarco/endoplasmic reticulum Ca^{2+}-ATPase 2 expression in diabetic nephropathy

Hengjiang Guo[1,#], Aili Cao[1,#], Shuang Chu[1], Yi Wang[1], Yingjun Zang[1], Xiaodong Mao[1], Hao Wang[2], Yunman Wang[2], Cheng Liu[3], Xuemei Zhang[4,*], Wen Peng[1,2,*]

[1]Laboratory of Renal Disease, [2]Department of Nephrology, Putuo Hospital, Shanghai University of Traditional Chinese Medicine, Shanghai, China

[3]Experimental Research Center, Putuo Hospital, Shanghai University of Traditional Chinese Medicine, Shanghai, China

[4]Department of Pharmacology, School of Pharmacy, Fudan University, Shanghai 201203, China

[#] These authors contributed equally to this work.

*** Correspondence:**
Wen Peng
pengwen_01@vip.sina.com
Xue-Mei Zhang
xuemzhang@fudan.edu.cn

1 Supplementary Figures and Tables

1.1 Supplementary Figures

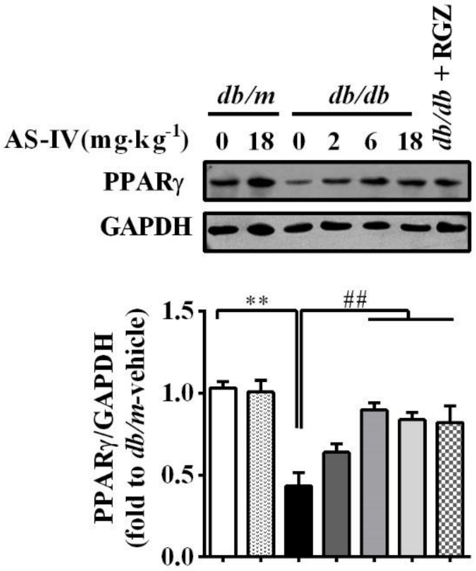

Supplementary Figure 1. AS-IV and RGZ restored PPARγ expression in renal cortex of *db/db* mice. 8-week-old *db/db* mice were treated with different doses of AS-IV or RGZ for 8 consecutive weeks. Data are expressed as mean ± SEM. n=3. $^{**}P < 0.01$; $^{\#\#}P < 0.01$.

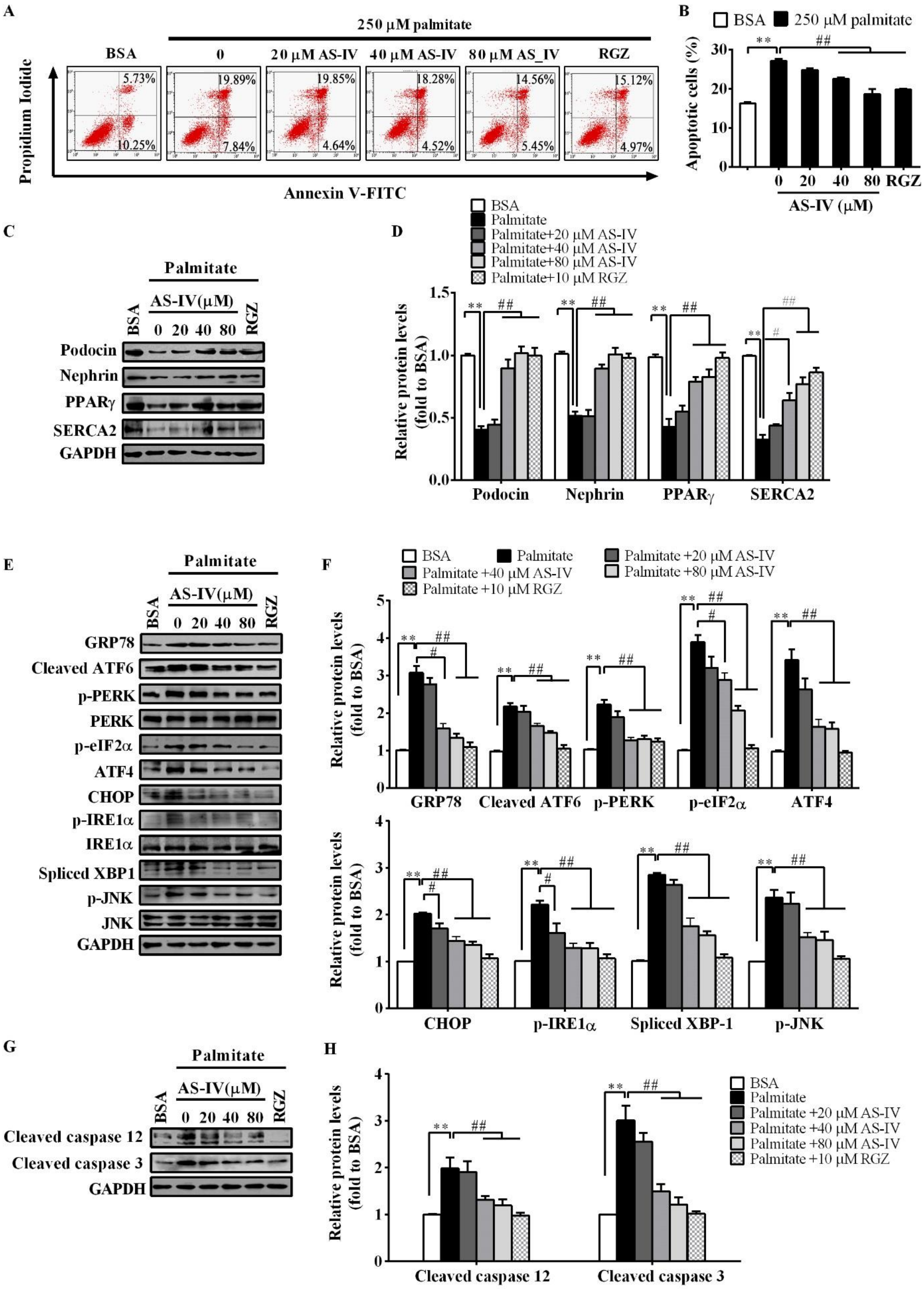
A
250 μM palmitate
BSA
0
20 μM AS-IV
40 μM AS-IV
80 μM AS_IV
RGZ
Propidium Iodide
Annexin V-FITC
5.73%
10.25%
19.89%
7.84%
19.85%
4.64%
18.28%
4.52%
14.56%
5.45%
15.12%
4.97%
B
BSA
250 μM palmitate
Apoptotic cells (%)
AS-IV (μM)
C
Palmitate
AS-IV(μM)
BSA
RGZ
Podocin
Nephrin
PPARγ
SERCA2
GAPDH
D
Palmitate
Palmitate+20 μM AS-IV
Palmitate+40 μM AS-IV
Palmitate+80 μM AS-IV
Palmitate+10 μM RGZ
Relative protein levels (fold to BSA)
E
GRP78
Cleaved ATF6
p-PERK
PERK
p-eIF2α
ATF4
CHOP
p-IRE1α
IRE1α
Spliced XBP1
p-JNK
JNK
F
Spliced XBP-1
G
Cleaved caspase 12
Cleaved caspase 3
H

Supplementary Figure 2. AS-IV and RGZ restored SERCA2 expression and inhibited ER stress-mediated apoptosis in palmitate-stimulated podocytes. Podocytes were pretreated with or without AS-IV or RGZ at the indicated concentrations for 12 h followed by 250 μM palmitate exposure for 24h. (A) Representative flow cytometry results for podocytes under different cultural conditions. (B) Semiquantitative data showing percentage of apoptotic podocytes under different cultural conditions. (C and D) Representative immunoblots (C) and quantification (D) of Podocin, Nephrin, PPARγ and SERCA2 under different conditions. (E and F) Representative immunoblots (E) and quantification (F) of ER stress markers under different conditions. (G and H) Representative immunoblots (G) and quantification (H) of apoptosis marker proteins under different conditions. Data are expressed as mean ± SEM. n=3. $^{**}P < 0.01$; $^{\#}P < 0.05$, $^{\#\#}P < 0.01$. One-way ANOVA and Newman-Keuls multiple comparisons test (B, D, F, H).

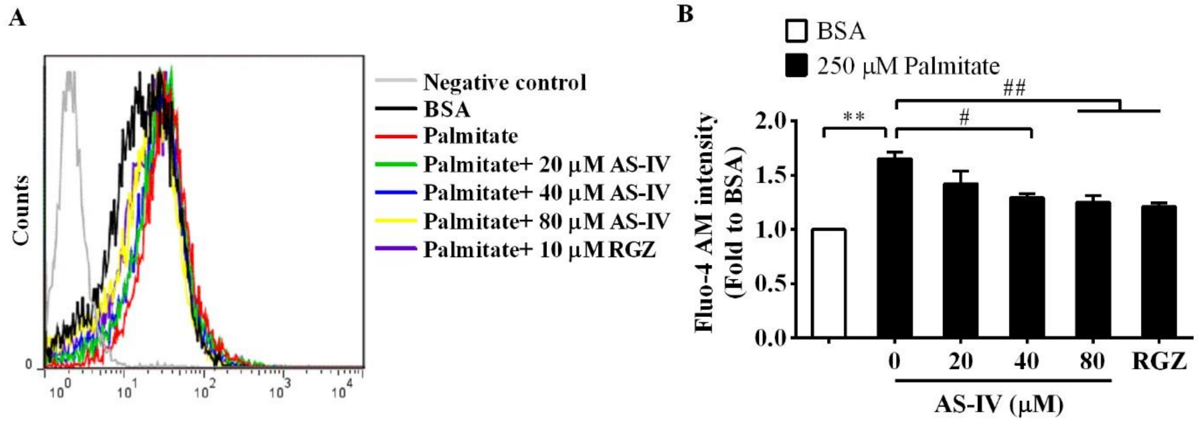

Supplementary Figure 3. AS-IV and RGZ improved intracellular Ca^{2+} homeostasis. Podocytes were pretreated with or without AS-IV or RGZ at the indicated concentrations for 12 h followed by 250 μM palmitate exposure for 24h, and then cytosolic Ca^{2+} were labeled with Fluo-4 AM and analyzed by flow cytometry. A. Representative picture of Fluo-4 AM staining showing intracellular Ca^{2+} levels under different conditions. (B) The relative fluo-4 AM fluorescence intensity under different conditions. n=3. $^{**}P < 0.01$; $^{\#}P < 0.05$, $^{\#\#}P < 0.01$. One-way ANOVA and Newman-Keuls multiple comparisons test (B).
